# The influence of flap design on the relevance of biomaterials in regenerative periodontal surgery

**DOI:** 10.1002/jper.70034

**Published:** 2025-12-10

**Authors:** Payvand Menhadji, Neha Kansagra, Luigi Nibali

**Affiliations:** ^1^ Centre for Host Microbiome Interactions Faculty of Dentistry Periodontology Unit, Oral & Craniofacial Sciences, King's College London London UK

**Keywords:** biomaterials, flap design, minimally invasive periodontal surgery, periodontal regeneration, single‐flap variants

## Abstract

**Background:**

The aim of this systematic review was to explore whether the benefits of biomaterials in periodontal regenerative/reconstructive surgery are affected by the type of flap employed. The study addresses the existing gap in the evidence to substantiate the beneficial effect of minimal invasive periodontal surgical approach compared to a conventional access flap (AF) surgery for the treatment of intrabony periodontal defects.

**Methods:**

Electronic search on Embase, Medline, Cochrane, Scopus, and manual search were performed to identify suitable randomized controlled trials on periodontal regenerative/reconstructive surgery. The Cochrane Collaboration's tool was used to assess the risk of bias. Papers were divided according to flap design into AFs, papilla preservation flaps (PPFs), and single‐flap variants (SFVs). Meta‐analyses were carried out to assess the effect of the added biomaterial compared with controls on probing pocket depth (PPD) and clinical attachment level (CAL), subdivided by flap design.

**Results:**

Of the 1311 papers initially identified, 523 underwent full‐text screening, and 29 publications representing 28 studies were ultimately included. In Group 1 (AFs, *n* = 6), the meta‐analysis revealed a statistically significant additional effect of biomaterials on improving CAL outcomes at 12 months, with an additional 2.46 mm CAL gain compared to control (95% confidence interval [CI]: 0.37–4.56, *p* = 0.02). In Group 2 (PPFs, *n* = 19), biomaterials provided a statistically significant benefit compared to PPF alone, with an additional 1.86 mm PPD reduction (95% CI: 0.29–3.42, *p* = 0.02) and 2.15 mm CAL gain (95% CI = 0.59–3.70, *p* = 0.01) at 12 months. In Group 3 (SFVs, *n* = 3), there was no statistically significant difference between the minimally invasive flap alone and its combination with biomaterials in terms of PPD reduction or CAL gain at 12 months.

**Conclusion:**

This systematic review suggests that SFVs may reduce the need for biomaterials due to their minimally invasive nature. The limited number of studies included in the SFV group and the high heterogeneity represent limitations of this review. It is still unclear whether flap design alone or the addition of biomaterials has a greater impact on clinical outcomes such as PPD reduction and CAL gain. The relative importance of biomaterials in periodontal regeneration requires further investigation.

**Plain language summary:**

This study reviewed existing clinical trials to understand whether the way gum tissue is lifted during surgery—known as flap design—affects the need for added materials (biomaterials) when treating bone loss caused by gum disease. Biomaterials are often used to support healing in regenerative periodontal surgery. However, newer surgical techniques that involve smaller trauma may reduce the need for these materials. The review grouped studies into 3 types of flap designs: traditional access flaps, papilla preservation flaps, and single‐flap variants (a minimally invasive technique). It found that using biomaterials improved outcomes when combined with traditional or papilla‐preserving flaps. In contrast, no significant benefit was observed when biomaterials were added to single‐flap variants. These findings suggest that with certain minimally invasive techniques, effective healing may occur even without biomaterials. However, only a few studies examined single‐flap variants, and results varied across studies, meaning that more research is needed. Understanding when biomaterials are truly necessary can help reduce treatment costs, improve healing, and guide better surgical choices for patients with advanced gum disease.

## 1 INTRODUCTION

Periodontal intraosseous defects (also called “intrabony” or “vertical” defects)^1^ are associated with a higher risk of progression in absence of treatment and, as such, are often considered to require surgical intervention beyond cause‐related periodontal therapy.^2^ Pioneering studies in the 1980s have shown that intraosseous defects have potential for healing through regeneration, including the formation of new attachment, regrowth of periodontal ligament and bone measurable clinically, radiographically, and histologically.^3^, ^4^, ^5^, ^6^ This has traditionally been achieved with the use of biomaterials, in the context of guided tissue regeneration (GTR) or with the use of enamel matrix derivatives (EMD), currently considered gold‐standard materials for periodontal regeneration.^7^

Development of periodontal regenerative medicine in the past 30 years has followed 2 distinctive, though totally interlaced paths. The interest of researchers has so far focused on regenerative materials and products on 1 side and on novel surgical approaches on the other side. In the area of the surgical approaches, clinical innovation in flap design and handling has radically changed surgery and has allowed a drastic limitation of inter‐dental wound failure from up to 100% with conventional flap approaches to < 10% with the more modern approaches.^8^, ^9^, ^10^, ^11^, ^12^, ^13^ Minimally invasive surgery^14^ and minimally invasive surgical techniques (MIST)^12^ are based on the preservation of interdental papilla associated with the minimal elevation of small buccal and lingual flaps. Single‐flap variants (SFVs) include the modified MIST (M‐MIST)^11^ and the single‐flap approach (SFA)^15^ which are characterized by a very limited elevation of the buccal side alone of the interdental papilla. Some evidence over the past 15 years^16^, ^17^, ^18^, ^19^ points toward substantial pocket reduction and clinical attachment level (CAL) gain when minimally invasive surgical approaches are applied in the treatment of intrabony defects, even without the use of regenerative biomaterials.^20^

Several studies have investigated the adjunctive use of EMD in MIST, reporting statistically significant improvements in recession (REC) and bone fill (BF) when compared to MIST alone. These findings highlight the regenerative potential of combining biologics with conservative flap designs.^21^ In contrast, the use of EMD in non‐surgical, flapless therapy has not demonstrated added clinical benefit compared to non‐surgical therapy alone, underscoring the importance of surgical access in achieving periodontal regeneration.^22^ Beyond EMD, biologic agents more broadly—including growth factors and cell‐based therapies—have been shown to enhance regenerative outcomes when used in conjunction with bone grafts (BGs) or barrier membranes. Combination therapies, such as BGs with biologics or BGs with resorbable membranes, have demonstrated superior clinical and radiographic results compared to monotherapies.^23^ Importantly, the specific type of BG or biologic agent used appears to influence treatment success in intrabody defects. These findings reinforce the need to consider both the surgical technique and the adjunctive materials employed when aiming to optimize regenerative outcomes.

Periodontal regenerative surgery in intrabony defects results in improved probing pocket depth (PPD) and CAL gain compared with open flap debridement (OFD), translating in high rates of tooth survival.^24^ Combination approaches (GTR and grafting, and EMD and grafting) appear more efficacious compared to monotherapy.^24^ Furthermore, Nibali et al. in 2020 concluded that EMD or GTR in combination with papilla preservation techniques should be considered the treatment of choice for residual pockets with deep (≤3 mm) intrabony defects.^7^ Periodontal regenerative surgery represents a predictable approach to obtain the outcome of pocket depth of ≤4 mm in the short term.^22^ Thus, there is a clinical and radiographic benefit of periodontal regenerative surgery versus access flap surgery.^7^, ^22^, ^24^ However, a meta‐analysis suggested no significant difference in treatment of intrabony defects between the minimally invasive surgery (MIS) plus biomaterials group and the MIS alone group, indicating that it is important to take costs and benefits into consideration when a decision is made about a therapeutic approach.^23^

Periodontal regenerative surgery has evolved substantially over the past few decades, particularly in the context of managing intrabony defects. Historically, OFD was the standard surgical approach, but it provided limited regenerative outcomes. Advances in biologics and surgical techniques have since demonstrated that regenerative procedures, including those incorporating GTR or EMD, can lead to significant improvements in PPD reduction and CAL gain. Several studies have reported average CAL gains of 2–3 mm and PPD reductions of similar magnitude when using regenerative materials in conjunction with surgical interventions. These gains translate into improved long‐term tooth survival and stability. Notably, combination therapies (e.g., GTR + grafts or EMD + grafts) have consistently outperformed monotherapies in both clinical and radiographic parameters. However, recent evidence, including the systematic review by Nibali et al. (2020), raises an important question: are these biomaterials always necessary, particularly when minimally invasive flap designs are employed? Some meta‐analyses suggest that MIS alone may achieve comparable outcomes to MIS with biomaterials, prompting a re‐evaluation of the necessity, cost‐effectiveness, and biological justification for adjunctive biomaterial use in modern periodontal surgery.

Overall, the effectiveness of these minimally invasive periodontal surgical procedures in regenerating intrabony defects without biomaterials versus with biomaterials remains to be clarified in terms of clinical performance and patient perception and the benefits when compared to more traditional flap approaches. Furthermore, there is currently an absence of robust direct evidence to substantiate the beneficial effect of minimal invasive periodontal surgical approach compared to a conventional access flap surgery for the treatment of intrabony periodontal defects.^25^

Consequently, we aim to explore whether the benefits of biomaterials in periodontal regenerative/reconstructive surgery are affected by the type of flap employed.

## 2 MATERIALS AND METHODS

A protocol was developed in adherence to the PRISMA‐P (Preferred Reporting Items for Systematic review and Meta‐Analysis Protocols) checklist based on Moher et al. 2009 and the AMSTAR checklist (Assessing the Methodological Quality of Systematic Reviews) by Shea et al. 2017.^26^, ^27^ This systematic review was registered on PROSPERO with registration number CRD42023398026.

### 2.1 Focused question

Broad review question: Is the clinical benefit of biomaterials in periodontal regenerative/reconstructive surgery (measured as probing depth reduction [PD reduction] and CAL gain) affected by the flap design employed (traditional flap vs. papilla preservation vs. SFVs)?

### 2.2 PICO(S)

1. Population: Adult human patients with periodontitis who have completed a cycle of non‐surgical periodontal therapy and present with intraosseous defects and associated residual pockets.
2. Intervention: Any type of regenerative/reconstructive surgery with GTR, EMD, growth factors, or combination.
3. Comparison: AF surgery, papilla preservation flap (PPF), or SFV with no biomaterials.
4. Outcomes
  ‐ Primary outcomes: PD reduction and CAL gain.
  ‐ Secondary outcomes: REC, bone gain, pocket “closure” (namely presence of PD at experimental site ≤ 4 mm at study follow‐up), tooth loss, number needed to treat (NNT), PROMs (patient‐reported outcome measures) and adverse events (AEs).
5. Studies: Randomized controlled trials.

### 2.3 Eligibility criteria

The inclusion criteria for studies in the systematic review are:

1. Study design: Randomized controlled trials investigating regenerative periodontal therapy with “SFVs” or “Papilla Preservation Flaps” or “Access Flaps” flap design for the treatment of intraosseous periodontal defects. Sub‐analyses on the efficacy of biomaterials.
2. Including at least a total of 20 patients in the whole study.
3. Reporting clinical outcomes of treatment (PPD, CAL, radiographic bone levels, tooth loss, and patient‐reported outcomes)
4. All included studies were required to report primary clinical outcomes (PPD and/or CAL) at 12 months; studies reporting only secondary or partial outcomes without primary endpoint data were deemed acceptable.
5. At least 12‐months follow‐up.
6. Written in English.

### 2.4 Definitions

Given the heterogeneity of periodontal regenerative surgical techniques, the following definitions were applied to this review:

1. Periodontitis: This will include staging and grading of periodontitis based on the 2017 world workshop classification^28^ or previous classifications.^29^
2. “Intrabony defects”: Defects with a base apical to the interdental alveolar crest, surrounded by 1, 2, or 3 bony walls or a combination with at least 3 mm of intrabony component.
3. Regenerative/reconstructive surgery: Any type of muco‐periosteal flap providing access to the root for debridement and applied barriers/growth factor/bone replacement materials (including GTR, EMD, growth factors, or combination) aimed to obtain the reconstruction of lost periodontium.
4. “Access Flap” (AF): Any type of mucoperiosteal flap providing access to the root for debridement followed by re‐positioning of the gingival at pre‐surgical level (alternative name: Open Flap Debridement).
5. “Papilla Preservation Flap” (PPF): Any flap employed for regenerative/reconstructive periodontal surgery not falling into the definition above (including flaps with papilla preservation).
6. “Single‐Flap Variant” (SFV): A flap where the papilla is not elevated or only 1 aspect of the papilla is elevated, including M‐MIST,^11^ SFA^15^ or its modifications,^30^ entire papilla preservation (EPP),^31^ non‐incised papillae surgical approach (NIPSA),^32^ or equivalent.

### 2.5 Information sources

The search was conducted through the electronic databases Embase, MEDLINE, Scopus, and Cochrane library and was complemented by a search through the reference lists of included studies. No language restriction was included in the initial search. Among published literature, peer‐reviewed studies, reports, book chapters, and conference abstracts were screened. Narrative or systematic reviews on the topic were searched in order to identify suitable papers. The search was complemented by searches on the Open Grey database and by a hand search in *Journal of Dental Research*, *Journal of Periodontology*, *Journal of Clinical Periodontology* and *Journal of Periodontal Research* (from 2001 to August 31, 2024).

### 2.6 Search strategy

A detailed description of the methods employed in this research is contained in Supporting Information in the online *Journal of Periodontology*.

### 2.7 Data extraction

Study selection was conducted by 2 independent reviewers (authors P.M. and N.K.) in the following stages:

1. Identify screening of potentially suitable titles and abstracts against the inclusion criteria to identify potentially relevant papers, resulting in a complete database by merging studies included by at least 1 reviewer;
2. Screening of the full papers identified as possibly being relevant from the initial screening.

Studies not meeting the inclusion criteria were excluded. In the presence of disagreement between reviewers, the decision regarding study eligibility was attempted by reviewers reaching a consensus. Where continued disagreement was apparent, an arbitrator (L.N.) judged the study inclusion.

The following data were extracted:

1. Number of patients included.
2. Patients’ demographics (age, ethnicity, sex, smoking).
3. Definition and diagnosis of periodontal cases included (e.g., aggressive periodontitis, chronic periodontitis, Stage, Grade).

### 2.8 Definition of intraosseous defects

1. Clinical methods (assessment and treatment).
2. Type of regenerative surgery.
3. Use of biomaterials.
4. Description of flap.
5. Length of follow‐up.
6. Number of patients lost to follow‐up.
7. Clinical outcomes.
8. Radiographic outcomes.
9. Patient‐reported outcomes.
10. Adverse events.

### 2.9 Quality assessment—risk of bias

The quality of the included studies was assessed through sensitivity analysis as it will impact on the overall results and conclusions.^33^ The Cochrane Collaborations tool will be used for assessing risk of bias.^34^

### 2.10 Data synthesis

Studies were summarized narratively by their chief characteristics. Results were stratified separately according to flap design (AF vs. PPF vs. SFV). A meta‐analysis was considered appropriate and was performed in the presence of a significant number of studies of similar design and judged of acceptable quality. In cases of high heterogeneity, efforts were made to identify the sources of heterogeneity. Random‐effects meta‐analyses of the selected studies were applied to account for methodological differences among studies. Separate analyses were conducted by type of regenerative surgery and length of follow‐up. Forest plots were produced, where appropriate, to graphically represent the differences in outcomes between groups using the patient as the unit of analysis. A *p*‐value of 0.05 was used as the level of significance. Heterogeneity was assessed with a chi‐squared test and the *I* ^2^ test. The suggested interpretation of I^2^ was: 0%–40% may represent low heterogeneity, 30%–60% moderate heterogeneity, 50%–90% substantial heterogeneity, and 75%–100% considerable heterogeneity.^34^ Funnel plots and Egger's test were used to explore the presence of publication bias.

Evidence provided by randomized controlled trials (RCTs) was rated using different levels of methodological strength, modified from GRADE (Grading of Recommendations Assessment, Development, and Evaluation).^35^ Three different strengths of evidence were considered:

1. High: At least 3 RCTs at low risk of bias and low heterogeneity.
2. Moderate: More than 1 RCT and at least 1 RCT at low risk of bias, low heterogeneity.
3. Low: Lack of RCT or RCT at high risk of bias or high heterogeneity.

## 3 RESULTS

### 3.1 Study selection

**FIGURE 1:**
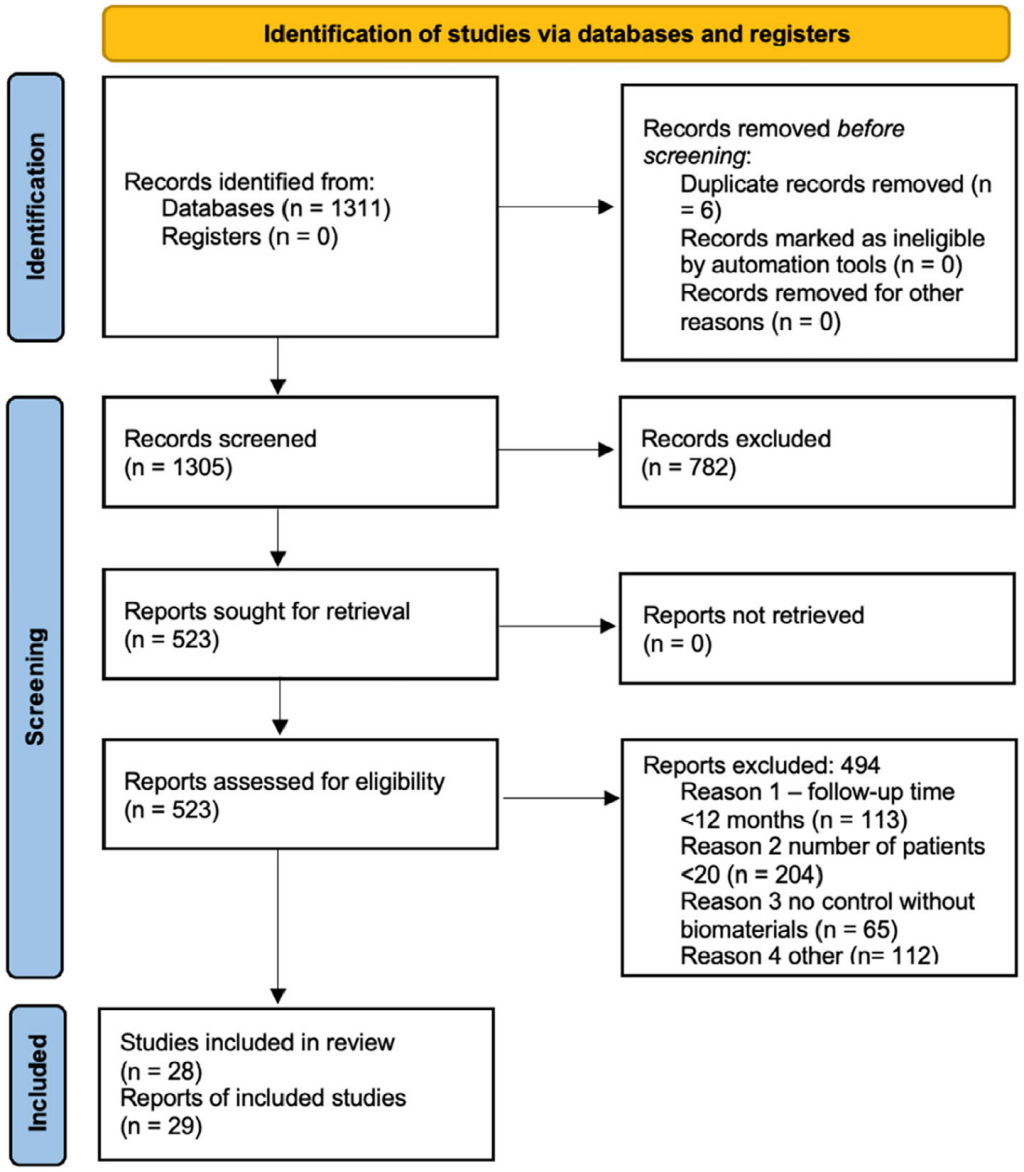
PRISMA flowchart—identification of studies.

Figure 1 presents the PRISMA flowchart for this review. The initial search identified 1311 papers across all databases, from which duplicates were removed. Following title and abstract screening, 782 articles were excluded and 523 articles were selected for full‐text review, with a 92.71% agreement between reviewers at step 1 (kappa score = 0.656) and 96.67% at step 2 (kappa score = 0.929) Of these, 29 articles were deemed eligible and included in the systematic review. Notably, these 29 articles represent 28 unique studies, as 1 study reported both 1‐year and 10‐year follow‐up results for the same population.^36^

#### 3.1.1 Study characteristics

**FIGURE 2:**
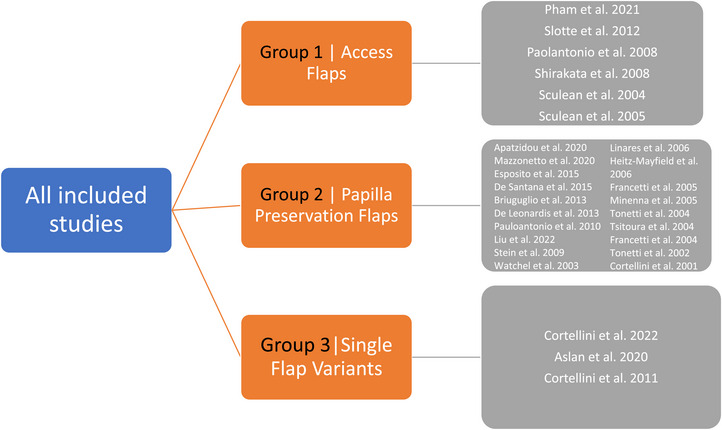
Included studies categorised into three groups—group 1 single flap variants, group 2 papilla preservation flaps, group access flaps.

Table 1 summarizes the characteristics of the 28 included studies (29 articles), which are visually represented in Figure 2. The papers were published between December 2001 and February 2024, and they all employed a RCT design. The studies were categorized into 3 groups: Group 1 consists of 6 studies focusing on AFs; Group 2 consists of 18 studies using PPFs using either simplified PPF,^37^ modified PPF,^8^ often as part of a MIST approach; and Group 3 consists of 3 studies focused on SFVs, including techniques such as the EPP and M‐MIST. The biomaterials utilized across the studies included enamel matrix derivatives, deproteinized bovine‐derived bone substitutes, and a range of resorbable membranes. Most studies were conducted in university settings, with follow‐up durations ranging from 12 months to 10 years, and all were published in English. Smokers were included in some studies.^38^

**TABLE 1:** Characteristics of included studies.

Study	Method	No. of patients	Age range (years)	Smoking	Country	Follow‐up	Flap design	Biomaterials
Pham (2021)^39^	Prospective, double‐masked RCT	30 8 (F) 22 (M)	32‐60	Smokers not included	Vietnam	1 year	1	**Control**: OFD only **Test 1**: PRF+OFD **Test 2**: GTR+RESORABLE MEMBRANE
Slotte et al. (2012)^40^	Double‐masked parallel‐group design, RCT	32 16 (F) 16 (M)	58.6 ± 3.7/58.1 ± 3.6	Smoking > 10 cigarettes per day excluded. 5 in control group, 3 in Test group.	Sweden	1 year	1	**Control**: OFD **Test**: OFD + BBM
Paolantonio et al. (2008)^41^	RCT	51 29 (F) 22 (M)	41‐62	Smokers were not included	Italy	1 year	1	**Control**: OFD **Test 1**: CS **Test 2**: CM
Shirakata et al. (2008)^42^	RCT	30 18 (F) 12 (M)	55.3 ± 7.5/51.5 ± 10.3	3 smokers in control group, 2 in test group)	Japan	1 year	1	**Control**: OFD **Test**: CPC
Sculean et al. (2005)^43^	Parallel, RCT	32 17 (F) 15 (M)	Not reported	Smokers if they smoked over 10 cigarettes. There was no smokers in this category.	Germany	1 year	1	**Control**: access flap **Test**: BDX Coll + GTR
Sculean et al. (2004)^44^	Prospective, RCT	42 Female and Male breakdown not reported	47 ± 14.5	(1 smoker in control, 1 smoker in Test 1, 2 smokers in Test 2)	Germany	5 years	1	**Control**: OFD **Test 1**: EMD **Test 2**: EMD+ GTR
Apatzidou et al. (2021)^45^	Prospective, RCT	27 18 (F) 9 (F)	20‐68	16 smokers included in the study (5 in control, 6 in Test 1, 5 in Test 2)	Greece	1 year	2	**Control**: Minimal access flap; No graft materials **Test 1**: Minimal access flap—transplantation of autologous a‐ BMSCs/autologous FPL/collagen **Test 2**: Minimal access flap—placement of autologous FPL/collagen
Mazzonetto et al. (2020)^46^	Prospective, double blind, RCT	60 41 (F) 19 (M)	<35 years of age	Smokers not included	Brazil	1 year	2	**Control**: AgP+CS **Test 1**: AgP+CS/EMD **Test 2**: CP+CS/EMD
Esposito et al. (2015)^47^	Multi‐center RCT of parallel design	97 52 (F) 45 (M)	21‐75	22 smokers in total (grouped in moderate and heavy smokers) (12 in control group, 10 in test)	Belgium, Italy, Poland, Portugal	1 year	2	**Control**: BG **Test**: OFD
de Santana et al. (2015)^48^	Prospective, split‐mouth, RCT	30 19 (F) 11 (M)	39‐66	Smokers not included	United States of America	1 year	2	**Control**: MPPF and OFD **Test**: MPPF, OFD and rhFGF‐2/HA
Briguglio et al. (2013)^49^	RCT	90 54 (F) 46 (M)	38‐68	Smokers not included	Italy	2 years	2	**Control**: OFD **Test**: HA and OFD
De Leonardis et al. (2013)^50^	Split‐mouth, prospective, RCT	36 21 (F) 15 (M)	30‐68	Smokers not included	Italy	2 years	2	**Control**: OFD **Test 1**: EMD **Test 2**: EMD + HA &b‐TCP
Paolantonio et al. (2010)^51^	RCT	42 22 (F) 20 (M)	38‐64	Smokers not included	Italy	1 year	2	**Control**: OFD **Test 1**: GTR/aCPRT
Stein et al. (2009)^52^	Prospective RCT	45 31 (F) 14 (M)	33‐69	Smokers not included	Germany	1 year	2	**Control**: OFD **Test 1**: BCC/ABS
Linares et al. (2006)^53^	Parallel group, RCT	121 72 (F) 49 (M)	34‐58	Smokers included < 20 cigarettes a day (21 in Test group, 18 in control group)	Italy, Holland, United States of America, Belgium	1 year	2	**Control**: OFD **Test 1**: GTR
Heitz‐Mayfield et al., (2006)^54^	Parallel group, multi‐center, RCT	116 70 (F) 46 (M)	38‐61	Smokers were included (20 in Test group and 19 in control group)	Germany, Greece, Italy, Switzerland, United States of America, Belgium, and Britain	1 year	2	**Control: PPF** **Test: PPF +BRG+ membrane**
Francetti et al. (2005)^55^	Prospective, multi‐center RCT	153 87 (F) 66 (M)	30‐70	Smokers included (21 in control group, 26 in Test group)	Italy	2 years	2	**Control**: SPPF **Test**: SPPF + EMD
Minenna et al. (2005)^56^	Prospective, parallel group, RCT	32 15 (F) 17 (M)	28‐64	Smokers included (6 in control group, 8 in Test group)	Italy and Spain	1 year	2	**Control**: OFD **Test 1**: OFD + PLA/PGA
Tonetti et al. (2004)^57^	Prospective parallel multi‐center RCT	124 76 (F) 48 (M)	49.5 ± 11.3/51 ± 10.5	<20 cigarettes a day included (20 in control group and 21 in Test group)	Italy, Britain United Kingdom, Switzerland, United States of America, Belgium, Greece, and Germany	1 year	2	**Control**: PPF **Test**: PPF + GTR/bone replacement
Tsitoura et al. (2004)^58^	Parallel group, multi‐center, RCT	166 95 (F) 71 (M)	48 ± 9	34 smokers included	United Kingdom	1 year	2	**Control**: OFD **Test**: OFD + EMD
Francetti et al. (2004)^59^	Parallel group, RCT	153 87 (F) 66 (M)	30‐70	<20 cigarettes a day were included; 5 in test group and 3 in control group	Italy	2 years	2	**Control**: SPP **Test**: SPP + EMD
Tonetti et al. (2002)^60^	Parallel group RCT	24 13 (F) 11 (M)	30‐66	<20 cigarettes a day were included. 31 smokers in both Test and Control groups	Italy	1 year	2	**Control**: SPPF **Test**: SPPF + EMD
Cortellini et al. (2001)^10^	Parallel group, multi‐center, RCT	113 77 (F) 36 (M)	46.6 ± 11.7	<20 cigarettes a day were included. 30 in Test group, 34 in control group.	United Kingdom, Switzerland, Italy, Belgium	1 year	2	**Control**: SPPF **Test**: SPPF + membrane
Wachtel et al. (2003)^61^	Prospective, split mouth design, RCT	11 8 (F) 3 (M)	28‐64	<10 cigarettes a day included, 6 smokers included in whole study	Germany	1 year	2	**Control**: Microsurgical access flap procedure **Test 1**: Microsurgical access flap procedure + EMD
Liu et al. (2022)^62^	Single blind, RCT	31 17 (F) 14 (M)	24‐63	Yes	China	1 year	2	**Control**: MIST ms‐alone **Test**: MIST ms‐BDMX+CM
Cortellini et al. (2022)^36^	Prospective longitudinal, parallel group, RCT	45 21 (F) 24 (M)	28–71	Smokers not included	Italy	10 years	3	**Control**: M‐MIST **Test 1**: M‐MIST+EMD **Test 2**: M‐MIST+EMD+BMDX
Aslan et al. (2020)^63^	Single‐center, parallel‐ group, RCT	30 12 (F) 18 (M)	21‐63	Smokers not included	Turkey	1 year	3	**Control**: EPP **Test 1**: EPP EMD + BS
Cortellini et al. (2011)^17^	Parallel group, RCT	45 21 (F) 24 (M)	34‐59	5 smokers included in the study (1 in control, 2 in Test 1, 2 in Test 2)	Italy	1 year	3	**Control**: M‐MIST alone **Test 1**: M‐MIST+EMD **Test 2**: M‐MIST+EMD‐BMDX

Abbreviations: A‐BMSCs, autologous bone marrow‐derived mesenchymal stem cells; ABS, Autologous bone substitute; AgP, aggressive periodontitis; BBM, bovine bone mineral; BCC, bovine collagen composite; BG, bone graft; BDX, bovine derived bone substitute; BRG, bone replacement graft; CM, collagen membrane; CPC, calcium phosphate cement; CPRT, combined periodontal regenerative technique; EMD, enamel matrix derivative; EPP, entire papilla preservation technique; FPL, fibrin/platelet lysate; GTR, guided tissue regeneration; HA, hyaluronic acid; M‐MIST, modified‐minimally invasive surgical technique; MPPF, modified papilla preservation flap; NG, not given; OFD, open flap debridement; PGA, polyglycolic acid; PLA, polylactic acid; PRF, platelet rich fibrin; RCT, randomized controlled trial; rhFGF‐2, recombinant human fibroblast growth factor; SPPF, simplified papilla preservation flap; β‐TCP, beta‐tricalcium phosphate.

#### 3.1.2 Population and treatment characteristics

Table 1 presents the demographic and clinical characteristics of the populations included in the analyzed studies. The sample sizes ranged from 20 to 166 participants, with the age of the study populations spanning from 21 to 75 years. Studies excluded individuals classified as heavy smokers, defined as those consuming 20 or more cigarettes per day, while light smokers who smoked 10 cigarettes or less were included in some studies (Tonetti et al. 1995).^38^ Periodontitis was characterized as advanced chronic periodontitis, with the presence of at least 1 intrabony defect ≥3 mm. Additionally, Table 2 outlines the characteristics of the surgical treatments, detailing the biomaterials used and the types of surgery performed (control and test groups). The studies were categorized based on the flap design definitions applied in this review into AF, PPF and SFV. Tsitoura et al. (2004) met the inclusion criteria; however, outcome data for the control group were not reported or utilized in the trial's analyses.^58^ As a result, the study was excluded from the meta‐analyses. Furthermore, Heitz‐Mayfield et al. (2006) reported CAL outcomes at 12 months; however, PPD data were not provided and, therefore, the study was excluded from the meta‐analysis for PPD.^54^

**TABLE 2:** Study results at 12 months.

Study	Flap design	Comparison (control vs. test)	CAL change control (mm) 12 months	CAL change test 1 (mm) 12 months	CAL change test 2 (mm) 12 months	PD reduction control (mm) 12 months	PD reduction test 1 (mm) 12 months	PD reduction test 2 (mm) 12 months
Pham (2021)^39^	1	OFD only PRF+OFD/GTR+resorable membrane/	3.37 (1.22)	5 (0.46)	4.53 (0.57)	3.37 (1.0)	4.8 (0.71)	4.63 (0.67)
Slotte et al. (2012)^40^	1	OFD/OF + BBM	2.8 (0.6)	2.3 (0.8)	n/a	4 (0.5)	3.2 (0.7)	n/a
Paolantonio et al. (2008)^41^	1	OFD/CS/CM	1.5 (0.2)	2.7 (0.1)	3.10 (0.3)	2.8 (0.1)	4.4 (0.6)	5.2 (0.5)
Shirakata et al. (2008)^42^	1	OFD/CPC	1.4 (0.8)	2.3 (1.0)	n/a	3.3 (1.2)	3.4 (1.2)	n/a
Sculean et al. (2005)^43^	1	Access flap /BDX Coll + GTR	1.9 (1.1)	4.1 (0.9)	n/a	3.6 (1.3)	5.4 (0.9)	n/a
Sculean et al. (2004)^44^	1	EMD/ EMD+ GTR/ OFD	1.6 (1.0)	3.4 (1.1)	3 (1.0)	3.3 1.1)	4.6 1.2)	4.4 (0.8)
Apatzidou et al. (2021)^45^	2	Group‐A: Minimal access flap—transplantation of autologous a‐ BMSCs/autologous FPL/collagen Group‐B: Minimal access flap—placement of autologous FPL/collagen Group‐C: Minimal access flap; no graft materials	3.1 (2.0)	2.8 (1.0)	3.1 (2.4)	4.2 (1.9)	3.4 (1.1)	3.5 (2.3)
Mazzonetto et al. (2020)^46^	2	AgP+CS/EMD AgP+CS CP+CS/EMD	1.6 (1.6)	2.4 (1.0)	2.10 (0.9)	1.9 (1.5)	2.3 (1.2)	2.5 (0.9)
Esposito et al. (2015)^47^	2	OFD/ BG	2.8 (0.3)	3.6 (0.3)	n/a	3.3 (0.3)	4.3 (0.3)	n/a
de Santana et al. (2015)^48^	2	MPPF and OFD/MPPF, OFD, and rhFGF‐2/HA	2.2 (0.5)	4.8 (0.2)	n/a	2.9 (0.9)	5.5 (1.4)	n/a
Briguglio et al. (2013)^49^	2	OFD alone/ HA	1.4 (1.8)	0.7 (0.9)	n/a	0.9 (1.3)	1.2 (0.6)	n/a
De Leonardis et al. (2013)^50^	2	EMD/EMD + HA &b‐TCP/ OFD	1.54 (0.64)	2.73 (0.64)	3.47 (0.65)	2.58 (0.55)	3.51 (0.58)	4 (0.42)
Paolantonio et al. (2010)^51^	2	OFD/ GTR/aCPRT	1.6 (0.01)	3.2 (0.3)	3.9 (0.1)	2.9 (0.1)	5.2 (0.2)	4.4 (‐0.1)
Stein et al. (2009)^52^	2	OFD/BCC/ABS	1.6 (0.7)	3 (0.8)	2.8 (0.9)	4.3 (0.8)	3.6 (0.7)	3.4 (0.8)
Linares et al. (2006)^53^	2	OFD/ GTR	2.5 (1.4)	3.5 (1.8)	n/a	3.2 (0)	3.7 (0.5)	n/a
Heitz‐Mayfield et al. (2006)^54^	2	PPF/ PPF + BRG+ membrane	2.5 (1.5)	3.3 (1.7)	NG	NG	NG	NG
Francetti et al. (2005)^55^	2	SPP/SPP + EMD	1.96 (0.18)	3.41 (0.14)	n/a	3 (‐0.17)	4 (0.13)	n/a
Minenna et al. (2005)^56^	2	OFD/OFD + PLA/PGA	3.4 (1.4)	3.6 (1.5)	n/a	3.9 (1.4)	4.6 (2.0)	n/a
Tonetti et al. (2004)^57^	2	PPF/PPF + GTR/bone replacement	2.5 (1.5)	3.3 (1.7)	n/a	3.2 (1.5)	3.7 (1.8)	n/a
Tsitoura et al. (2004)^58^	2	OFD/OFD + EMD	NG	3.2 (1.6)	NG	NG	NG	NG
Francetti et al. (2004)^59^	2	SPPF/SPPF + EMD	2.29 (0.95)	4.14 (1.35)	n/a	2.57 (1.27)	4.71 (1.6)	n/a
Tonetti et al. (2002)^60^	2	SPPF or MPPF/SPPF or MPPF + EMD	2.5 (1.5)	3.1 (1.5)	n/a	3.3 (1.7)	3.9 (1.7)	n/a
Cortellini et al. (2001)^10^	2	SPPF/SPPF + membrane	2.6 (1.8)	3.5 (2.1)	n/a	3.6 (2.1)	4.4 (2.4)	n/a
Wachtel et al. (2003)^61^	2	Microsurgical access flap procedure/microsurgical access flap procedure + EMD	1.7 (1.4)	3.6 (1.6)	n/a	2.1 (1.1)	3.9 (1.4)	n/a
Liu et al. (2022)^62^		MIST ms‐alone/ms‐ BMDX + CM	2.5 (1.8)	2.0 (1.4)	n/a	2.5 (1.2)	2.3 (1.5)	n/a
Cortellini et al. (2022)^36^	3	M‐MIST/M‐MIST+EMD/M‐MIST+EMD+BMDX	4.1 (1.4)	4.1 (1.2)	3.7 (1.3)	4.4 (1.6)	4.4 (1.2)	4 (1.3)
Aslan et al. (2020)^63^	3	EPP/ EPP EMD + BS	5.83 (1.12)	6.3 (2.5)	n/a	6.2 (1.33)	6.5 (2.65)	n/a
Cortellini et al. (2011)^17^	3	M‐MIST alone/M‐MIST+EMD/M‐MIST+EMD‐BMDX	4.1 (1.4)	4.1 (1.2)	3.7 (1.3)	4.4 (1.0)	4.4 (0.3)	4 (0.6)

Abbreviations: a‐BMSCs, autologous bone marrow–derived mesenchymal stem cells; AgP, aggressive periodontitis; BBM, bovine bone mineral; BDX, bovine‐derived xenograft; BG, bone graft; BMDX, bone mineral derivative xenograft; CAL, clinical attachment level; CM, collagen membrane; CPC, calcium phosphate cement; CPRT, combined periodontal regenerative technique; CS, calcium sulphate; EMD, enamel matrix derivative; EPP, entire papilla preservation technique; FPL, fibrin/platelet lysate; GTR, guided tissue regeneration; HA, hyaluronic acid; M‐MIST, modified‐minimally invasive surgical technique; MPPF, modified papilla preservation flap; OFD, open flap debridement; PD, probing depth; PGA, polyglycolic acid; PLA, polylactic acid; PRF, platelet‐rich fibrin; rhFGF‐2, recombinant human fibroblast growth factor; SPP, simplified papilla preservation; β‐TCP, beta‐tricalcium phosphate.

### 3.2 Meta‐analysis

**FIGURE 3:**
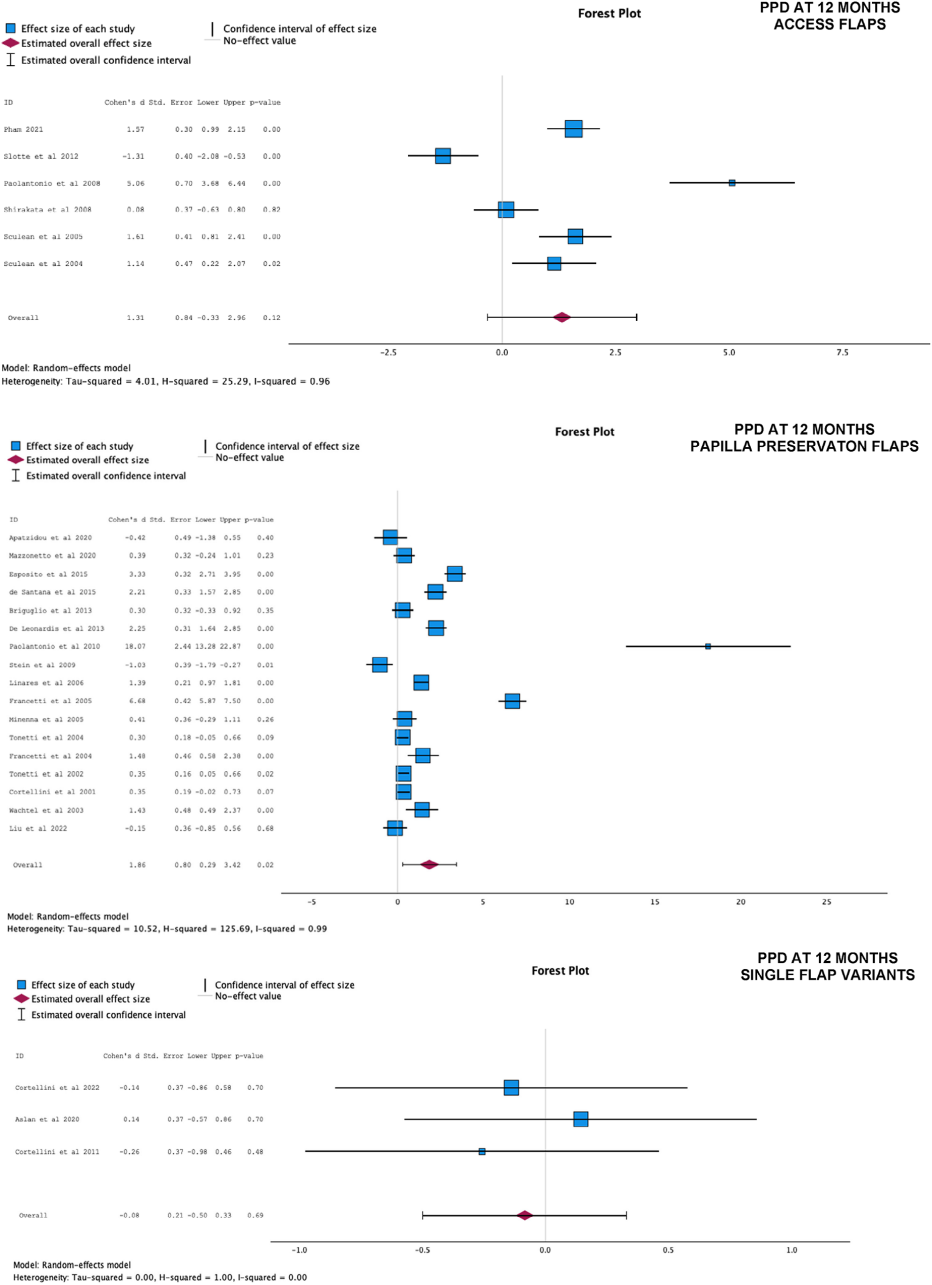
Forest plots for access flaps, papilla preservation flaps, single‐flap variants. Pocket probing depths at 12 months.

**FIGURE 4:**
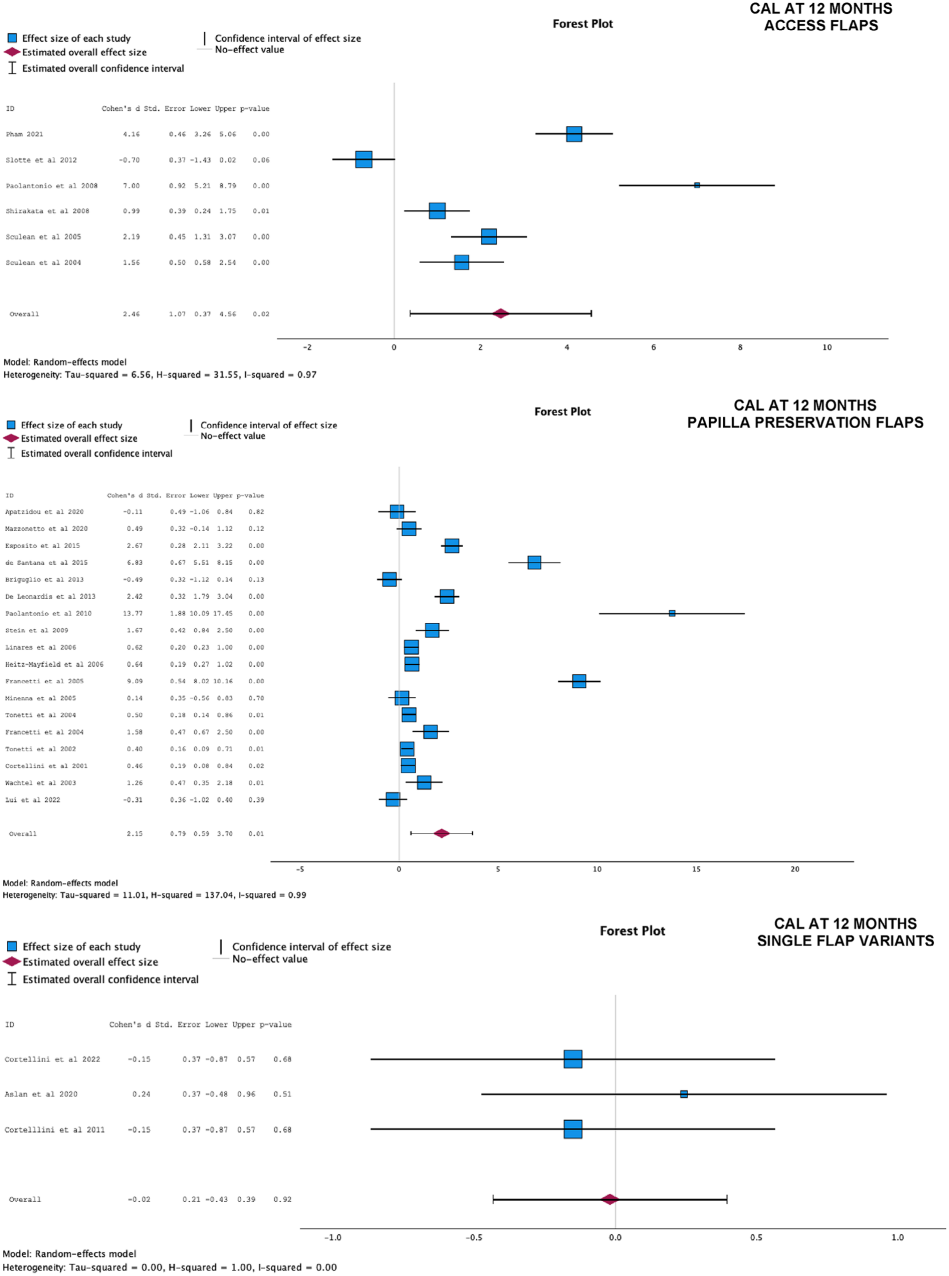
Forest plots of access flaps, papilla preservation flaps, and single‐flap variants. Clinical attachment levels at 12 months.

Figures 3 and 4 present the results of the meta‐analyses for PPD and CAL at 12 months, categorized by different flap designs. The analyses compared the effect of using differing flap designs with biomaterials (test groups) versus differing flap designs with no biomaterial (control groups):

- In Group 1 (AFs) (*n* = 6), the meta‐analysis revealed a statistically significant additional effect of biomaterials in improving CAL outcomes at 12 months (additional 2.46 mm CAL gain compared with control, 95% CI = 0.37;4.56, *p* = 0.02), but with high heterogeneity (*I* ^2 ^= 0.97). For PPD at 12 months, the studies favored of the use of biomaterials; however, the overall combined effect was not statistically significant (additional 1.31 mm PPD reduction compared with control, 95% CI = −0.33;2.96, *p* = 0.12), and with high heterogeneity (*I* ^2 ^= 0.96).
- In Group 2 (PPFs) (*n* = 19), the meta‐analyses demonstrated a statistically significant benefit when biomaterials were used alongside PPF techniques, compared with controls (PPF alone) for both PPD reduction (1.86 mm, 95% C.I. 0.29;3.42, *p* = 0.02) and CAL gain at 12 months (2.15 mm, 95% CI = 0.59–3.70, *p* = 0.01), both with high heterogeneity (*I* ^2 ^= 0.96 and 0.99, respectively).
- In Group 3 (SFVs) (*n* = 3), there was no statistically significant difference between the use of the minimally invasive flap alone and its combination with biomaterials in terms of PPD (*p* = 0.69) and CAL at 12 months (*p* = 0.92).

### 3.3 Funnel plots

**FIGURE 5:**
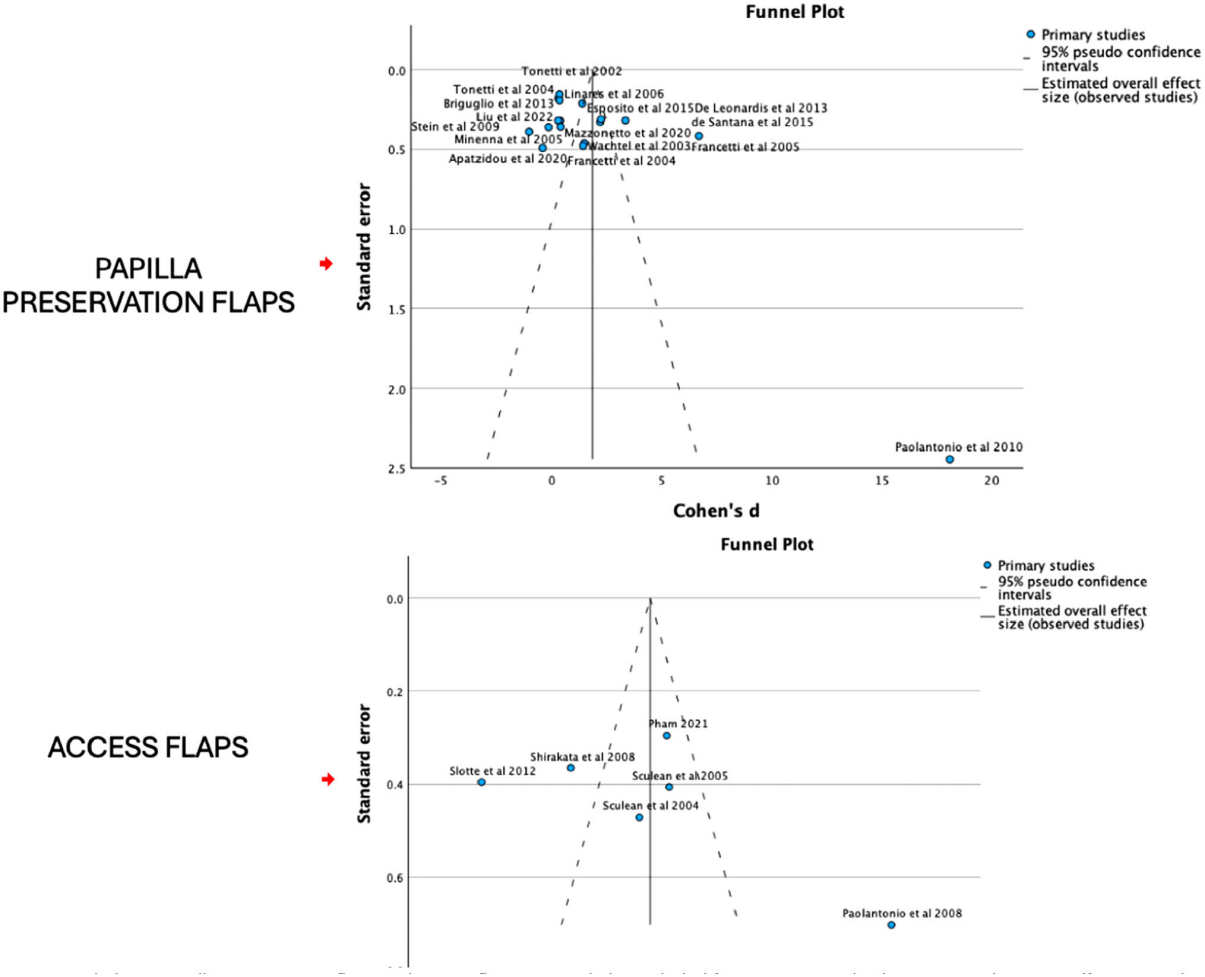
Funnel plots of papilla preservation flaps and access flaps. Funnel plot included for Group 1. Single‐flap variants due to insufficient number of studies.

As shown in Figure 5, both funnel plots show that most studies align well with the expected overall effect size, indicating consistency and minimal variability in the results. The majority of studies fall within the 95% pseudo confidence intervals, suggesting reliable findings. However, in both plots, an outlier (Paolantonio et al. 2010 in 1, and Paolantonio et al. 2008 in the other) appears far to the right, indicating a much larger effect size and potentially pointing to bias or unique study conditions.

### 3.4 Risk of bias analysis

Table 3 provides an overview of the risk of bias assessment conducted using the Cochrane Collaboration's tool. This systematic review carefully evaluated potential biases across the included studies, recognizing the variability in study quality. Of the 28 studies, 7 were classified as low risk, 15 were identified as having low to moderate risk, and 4 studies exhibited at least 1 high‐risk element.

**TABLE 3:** Risk of bias analysis.

Reference	Random sequence generation (selection bias)	Allocation concealment (selection bias)	Blinding of participants and personnel (performance bias)	Blinding of outcome assessment (detection bias)	Incomplete outcome data (attrition bias)	Selective reporting (reporting bias)
Cortellini et al. (2022)^36^						
Aslan et al. (2020)^63^						
Cortellini et al. (2011)^17^						
Apatzidou et al. (2021)^45^						
Mazzonetto et al. (2020)^46^						
Esposito et al. (2015)^47^						
de Santana et al. (2015)^48^						
Briguglio et al. (2013)^49^						
De Leonardis et al. (2013)^50^						
Paolantonio et al. (2010)^51^						
Stein et al. (2009)^52^						
Linares et al. (2006)^53^						
Francetti et al. (2005)^55^						
Minenna et al. (2005)^56^			Not blinded			
Tonetti et al. (2004)^57^						
Tsitoura et al. (2004)^58^						
Francetti et al. (2004)^59^						
Tonetti et al. (2002)^60^						
Cortellini et al. (2001)^10^						
Wachtel et al. (2003)^61^						
Pham (2021)^39^						
Slotte et al. (2012)^40^						
Paolantonio et al. (2008)^41^						
Shirakata et al. (2008)^42^						
Sculean et al. (2005)^43^						
Sculean et al. (2004)^44^						

Red boxes indicate high risk of bias, warranting cautious interpretation due to potential methodological flaws (e.g., inadequate blinding or allocation concealment). Green boxes represent low risk and greater data reliability. Amber boxes reflect unclear risk due to insufficient information, introducing uncertainty in study validity.

## 4 DISCUSSION

This systematic review suggests that in periodontal regenerative surgery, biomaterials are of more critical importance for improving clinical outcomes when used with traditional flap designs. While the addition of biomaterials improved clinical outcomes at 12 months alongside AFs or PPFs, no such effect was observed when single‐flap approaches were employed. This suggests that, while biomaterials play a crucial role in enhancing the effectiveness of traditional flap surgeries, as the flap elevation is minimized, the addition of biomaterials may become less relevant for clinical outcomes.

Biomaterials used in the studies included enamel matrix derivative, deproteinized bovine bone mineral, hyaluronic acid, calcium phosphate cement, resorbable collagen membranes, and recombinant human fibroblast growth factor. Biomaterials were either used in the defects as a sole therapy or in combinations of the above. The combination of biomaterials and modern flap techniques like PPFs yields optimal results: In both Group 1 (AF) and Group 2 (PPFs), the combination of PPFs with biomaterials produced improvements in both PPD reduction (in the range of additional 1.3–1.8 mm compared with controls) and CAL gain (over 2 mm compared with controls) at 12 months (statistically significant for all comparisons except PPD change in the AF group). This suggests that PPFs, which are designed to preserve soft tissue stability, benefit from the adjunctive use of biomaterials, offering superior regenerative outcomes.

Minimally invasive techniques may reduce the reliance on biomaterials: In Group 1 (SFVs), no statistically significant difference was found between using biomaterials with SFVs and using this flap design alone. Two out of 3 studies in this group actually favored the use of SFV alone, suggesting that the inherent surgical stability of minimally invasive flap designs may be sufficient to promote healing without the need for additional biomaterials.^17^, ^36^

Minimally‐invasive surgery (especially SFVs) are able to utilize a blood clot alone to regenerate the intrabony defect and question the benefit of adding biomaterials. It may be speculated that the increased flap stability with minimally‐invasive surgery makes the adjunctive use of biomaterials redundant. Many of the published reports in the past decade have evaluated MIST for the treatment of intrabony periodontal defects which involves a flap design with minimal incision and elevation that is adequate enough to access the defect under magnification. Such flap designs are thought to have the advantage of reduced surgical trauma and creation of a contained surgical wound that preserves the blood clot, all of which could result in an early and enhanced wound healing. Similarly, the SFA technique first reported in 2007 by Trombelli^15^ involved elimination of bilateral flap reflection, when pockets were confined to either the buccal or palatal side, thus eliminating the involvement of non‐diseased sites. Trombelli et al. in 2021 concluded that SFVs without addition of EMD resulted in a significant clinical benefit to the procedure, thus questioning the adjunctive benefit of the biomaterial.^64^ This aligns with the European Federation of Periodontology (EFP) guidelines which recommend limiting flap elevation under some specific circumstances to optimize wound stability and reduce morbidity.^65^

It is important to note that the SFV group only included 3 studies, making the conclusion less robust and raising questions about a potential lack of power to observe differences between groups. This review's strict inclusion criteria—requiring at least 12 months of follow‐up and a minimum of 20 patients—may have further restricted the number of eligible studies. For example, several studies from the Trombelli series on the SFA, which could have provided valuable insights, were excluded for being retrospective studies, thus not meeting our RCT requirement.^66^, ^67^, ^68^ However, the direction of the results in the meta‐analyses related to this flap design seems to clearly point out toward a lack of effect, rather than to a lack of power.

In Group 2, which focused PPFs, our analysis revealed a statistically significant positive effect for the test groups, with substantial improvements in CAL gain and PPD reduction at the 12‐month follow‐up. These findings are consistent with previous research by Nibali et al. (2020), which demonstrated that the use of EMD and GTR, when combined with PPF techniques, significantly improved CAL gain and reduced PPD compared to access flap procedures alone.^7^ It's important to note that there is a lot of overlap of included studies between both of these systematic reviews, which may account for the consistency in findings. In our Group 2 studies, although papilla preservation techniques were used, some procedures involved larger flaps and vertical releasing incisions. In these cases, biomaterials likely played a more critical role in enhancing regeneration. However, with the evolution of PPFs, these techniques are being employed in an even more minimally invasive manner, which may reduce the reliance on biomaterials for achieving optimal clinical outcomes. The larger number of studies included in this group provided a stronger evidence base, allowing for more definitive conclusions. However, due to missing data on PPD and CAL gain at the 12‐month mark, we were unable to include Tsitoura et al. (2004) in the meta‐analysis.

One limitation of this systematic review is the significant heterogeneity among the included studies, particularly in terms of study design, sample size, biomaterials used and flap designs. This variability led to a high heterogeneity in the meta‐analyses, making it challenging to draw definitive conclusions. Additionally, heterogeneity in follow‐up periods, inclusion of smokers, biomaterial selection, and population demographics such as ethnicity further limited comparability across studies. Another limitation is the inclusion of some studies demonstrating moderate‐high risk of bias, as highlighted in Table 3, which affects the overall strength of the evidence, although the majority were deemed as low risk.

To specifically evaluate the impact of flap design on the clinical effectiveness of biomaterials, we included only studies that incorporated a non‐biomaterial control group within the same flap design. This approach enabled direct within‐study comparisons, reducing the influence of confounding variables such as defect morphology, patient characteristics, and surgical technique. While this criterion enhanced internal validity and allowed a focused analysis of effect modification by flap design, it also led to the exclusion of several RCTs that did not include a non‐biomaterial comparator, thereby reducing the overall number of studies included, particularly in Groups 1 and 3.

Additionally, although advanced statistical approaches such as mixed‐effects or network meta‐analyses can provide broader comparative insight, the limited number of studies within each flap subgroup and the heterogeneity of protocols and materials precluded the reliable use of such models. Pairwise meta‐analysis with random‐effects models was therefore selected to ensure clinical interpretability and methodological consistency. We acknowledge this trade‐off and highlight the need for future studies, including those with individual patient‐level data, to further explore the nuanced interactions between flap design, biomaterial use, and regenerative outcomes.

This review has several strengths that enhance its credibility. It followed a comprehensive and rigorous methodology, adhering to PRISMA guidelines and employing robust tools such as the Cochrane Risk of Bias for quality assessment. The broad and systematic search across multiple databases reduced the risk of missing relevant studies, and the inclusion of a well‐defined patient population ensured clinical relevance. The meta‐analyses included stratification based on different flap designs, offering valuable insights into how surgical techniques affect the outcomes of biomaterial use. By focusing on key clinical outcomes and conducting subgroup analyses, the review provided practical, actionable findings for clinicians. Additionally, the thorough assessment of bias and heterogeneity, as well as the use of random‐effects models, strengthened the validity of the overall findings. Although most studies align well with the expected overall effect size as observed in funnel plots, we cannot exclude the possibility of some publication bias.

Future studies should aim to include a broader range of clinical trials that specifically evaluate SFVs used in isolation, to assess their effectiveness in periodontal regeneration without the adjunct of biomaterials. By focusing on SFVs alone, such studies can help clarify their benefits over traditional approaches, particularly in terms of wound stability, faster healing, and CAL gain. These studies should also prioritize long‐term follow‐ups and include various defect types and patient populations to enhance the generalizability of the findings. Expanding the scope of included trials will increase the statistical power of meta‐analyses and provide a more comprehensive evaluation of clinical outcomes, especially for newer, less‐studied flap designs such as EPP and NIPSA.^31^, ^32^ Standardizing outcome measures, conducting long‐term follow‐up studies, and including patient‐centered outcomes will also help clarify the most effective approaches for periodontal regeneration. This will ensure that clinicians can make informed decisions regarding the optimal combination of flap design and biomaterials for each patient. Although RCTs were included in this review, the high heterogeneity, inconsistency, and variability in results lead us to assess the level of evidence as low to moderate (GRADE). These factors reduce our confidence in the effect estimate and suggest that additional, more consistent research may be necessary to draw definitive conclusions.

## 5 CONCLUSION

This systematic review indicates that flap design may influence the clinical benefit of biomaterials in regenerative periodontal surgery. While biomaterials improved outcomes when used with AFs and PPFs, no significant added benefit was observed with SFVs, suggesting that minimally invasive approaches may reduce the need for adjunctive materials. However, the relative impact of flap design versus biomaterials remains unclear, and further high‐quality studies are needed. Flap and biomaterial selection should be guided by defect anatomy, surgical objectives, and individual patient factors.

## AUTHOR CONTRIBUTIONS

All authors have made substantial contributions to the conception and design of this systematic review. Payvand Menhadji and Neha Kansagra have been involved in data collection and data analysis. All authors have been involved in data interpretation, drafting the manuscript, and revising it critically and have given final approval of the version to be published.

## CONFLICT OF INTEREST STATEMENT

The authors declare no conflicts of interest in connection with this article.

## Supporting information



Supporting Information

## Data Availability

The data that support the findings of this study are available from the corresponding author upon reasonable request.
