# Supplementary material for: The influence of flap design on the relevance of biomaterials in regenerative periodontal surgery
Source: J Periodontol. 2025 Dec 10;97(4):697–719. doi: 10.1002/jper.70034 (PMC13169481; doi:10.1002/jper.70034)
Supplement: Supplementary file 1 — Supporting Information [file JPER-97-697-s001.docx]

**Supplementary File:**

A comprehensive electronic search was conducted using this Search Strategy:

**(((((((((((((((((((Open Flap for Debridement) OR (Access Flap)) OR (Open Flap Curettage)) OR (Modified Widman Flap)) OR (Open Clean-out)) OR (Guided Tissue Regeneration)) OR (Guided-Tissue-Regeneration)) OR (Periodontal Regeneration)) OR (GTR)) OR (Amelogenin)) OR (Emdogain)) OR (Enamel Matrix Derivative)) OR (Enamel Matrix Protein)) OR (Dental Enamel Protein)) OR (Growth Factors)) OR (Platelet Derived Growth Factors)) OR (Bone Graft)) OR (Autogenous Bone Graft)) OR (Allogenic Bone Graft) AND ((randomizedcontrolledtrial[Filter]) AND (humans[Filter]))) AND (((((((((((((((((((Periodontal pocket/therapy[MeSH Terms]) OR (Periodontal pocket/therapy) ) OR (Periodontal surger*)) OR (Periodontal adj2 surger*)) OR (Periodontal pocket/surgery[MeSH Terms])) OR (Periodontal pocket/surgery)) OR (Minimally Invasive Periodontal Surgery"[MeSH Terms])) OR (Minimally Invasive Periodontal Surgery) ) OR (Minimally-Invasive Surgery[MeSH Terms])) OR (Minimally-Invasive Surgery)) OR (Alveolar Bone Loss/surgery[MeSH Terms])) OR (Alveolar Bone Loss/surgery)) OR (Alveolar Bone Loss/therapy[MeSH Terms])) OR (Alveolar Bone Loss/therapy)) OR (Intra bony defect*)) OR (Infra bony defect*)) OR (Intrabony defect*)) OR (Infrabony defect*) AND ((randomizedcontrolledtrial[Filter]) AND (humans[Filter]))) - Saved search** Filters: **Randomized Controlled Trial, Humans** Sort by: **Most Recent**

**PRISMA FLOWCHART**

A protocol was developed in adherence to the PRISMA-P checklist (Preferred Reporting Items for Systematic review and Meta-analysis Protocols) based on Moher et al. 2009 and the AMSTAR checklist (Assessing the Methodological Quality of Systematic Reviews) by Shea et al. 201.

**Identification of studies via databases and registers**

Records removed *before screening*:

Duplicate records removed (n = 6)

Records marked as ineligible by automation tools (n = 0)

Records removed for other reasons (n = 0)

Records identified from:

Databases (n = 1311)

Registers (n = 0)

**Identification**

Records screened

(n = 1305)

Records excluded

(n = 782)

Reports sought for retrieval

(n = 523)

Reports not retrieved

(n = 0)

**Screening**

Reports assessed for eligibility

(n = 523)

Reports excluded: 494

Reason 1 – follow-up time <12 months (n = 113)

Reason 2 number of patients <20 (n = 204)

Reason 3 no control without biomaterials (n = 65)

Reason 4 other (n= 112)

Studies included in review

(n = 28)

Reports of included studies

(n = 29)

**Included**
